# Immune Sculpting of Norepinephrine on MHC-I, B7-1, IDO and B7-H1 Expression and Regulation of Proliferation and Invasion in Pancreatic Carcinoma Cells

**DOI:** 10.1371/journal.pone.0045491

**Published:** 2012-09-19

**Authors:** Liancai Wang, Han Liu, Xiangli Chen, Min Zhang, Keping Xie, Qingyong Ma

**Affiliations:** 1 Department of Hepatobiliary Surgery, First Affiliated Hospital of Xi’an Jiaotong University, Xi’an, Shaanxi Province, China; 2 Henan Province People’s Hospital, Zhengzhou, Henan Province, China; 3 Department of Gastrointestinal Medical Oncology, the University of Texas, MD Anderson Cancer Centre, Houston, Texas; University of Nebraska Medical Center, United States of America

## Abstract

**Background:**

The sympathetic neurotransmitter Norepinephrine (NE) contributes to tumorigenesis and cancer progression. This study aims to investigate the role of NE in modulating the immune phenotype and allowing pancreatic carcinoma (PC) cells to escape the immune response.

**Methods:**

Varied concentrations of NE and interferon-gamma (IFN-γ) were administrated to MIA PaCa-2 and BxPC-3 cell lines for 48 hours. Proliferation and invasion were then investigated using an MTT assay and a membrane invasion culture system respectively. MHC-I, B7-1, IDO and B7-H1 expression were measured using real-time quantitative RT-PCR, western blotting and immunocytochemistry. The synergistic and time-dependent effects of NE/IFN-γ were also investigated. Adrenergic antagonists were used to identify the relevant target receptor of NE.

**Results:**

The results showed that NE had dose-dependent and time-dependent effects on cell biological processes as well as on the expression of MHC-I, B7-1, IDO and B7-H1. These effects occurred mainly via the β_2_-adrenergic receptor. Long-term NE treatment was able to antagonize some of the effects of IFN-γ (after 2 weeks of treatment), but NE and IFN-γ had significant synergistic stimulatory effects on IDO and B7-H1 expression. The residual effects on biological activities lasted for 2 weeks, while the immunophenotypic changes decreased at early time points after treatment.

**Conclusions:**

NE plays important roles in modulating PC cell biological activities and affecting MHC-I, B7-1, IDO and B7-H1 expression in vitro, mainly via the β2-adrenergic receptor (β2-AR) in a time- and dose-dependent fashion. Only at extended treatment durations could NE affect PC cell progression and immune evasion.

## Introduction

Pancreatic carcinoma (PC) is still an incurable disease with a 5-year survival rate of less than 5%. It is evident that within the tumor microenvironment, there is often a loss of functional dendritic cells (DC) and lymphocytes [1]. In this case, the tumor cells have acquired the ability to impair host antitumor immunity, thus rendering the immune system incapable of effectively mediating tumor regression.

Fortunately, some encouraging progress has been made in the study of how tumor cells modify their immunogenic phenotype to allow immune escape [2], which is a key component of clinical prognosis because it affects tumor progression, allows invasion and metastasis, and impacts the therapeutic response. Recent studies have provided compelling evidence that tumor cells either build a microenvironment locally or reach other organs systemically by changing their immunogenic phenotypes [3], [4], particularly the expression levels of the major histocompatibility complex I (MHC-I), B7-1, indoleamine 2, 3-dioxygenase (IDO) and B7-H1.

Among the factors involved in tumor immune escape mechanisms that have been described to date, MHC-I and B7-1 are critical to the tumor development process due to their roles in antigen presentation to T-lymphocytes, activation of the T cell anti-tumor immune response and the regulation of natural killer (NK) cell function. The expression of these proteins is absent or low in some human tumors, and enhancing their expression may improve antitumor immunotherapy [5], [6].

IDO, an enzyme of the kynurenine pathway of tryptophan catabolism, catalyzes the initial and rate-limiting step of the pathway, which consists of the oxidative cleavage of the pyrrole ring of the indole nucleus of L-Trp to yield N-formylkynurenine [7]. Emerging evidence suggests that during cancer progression, activation of the IDO pathway might act as a preferred nodal modifier pathway for immune escape. Various human cancers, including PC, exhibit elevated expression of IDO, which plays a role in tumor immunoediting by establishing peripheral tolerance to tumor antigens [8]. Recent preclinical studies indicate that small molecule inhibitors of IDO are able to thwart the enzyme-mediated immunoediting process and improve the efficacy of chemotherapy [9].

B7-H1, an important co-suppressive molecule expressed on tumor-infiltrating macrophages and dendritic cells, as well as on tumor cells, contributes to immune evasion and facilitates tumor growth [10]. Programmed death-1 (PD-1) is the cognate receptor for B7-H1. Via the B7-H1/PD-1 pathway, B7-H1 can suppress T cell generation and activation as well as down-regulate the synthesis and secretion of IL-2 and IFN-γ by myeloid DCs and T cells [11]. B7-H1^+^ tumor cell lines can also induce apoptosis of immune effector cells, impairing the lethal effects of cytotoxic lymphocyte (CTL) [12]. Blocking B7-H1 could assist cancer immunotherapy [13].

In our previous clinical studies, we observed that MHC-I, B7-1, IDO and B7-H1 were expressed in PC, and combined IDO/B7-H1 or B7-1/B7-H1 [14] expression was shown to act as an independent prognostic marker for PC. Furthermore, B7-1, IDO and B7-H1 expression varied among different grades of PC malignancies and were higher in the tumor margin than in the central area of the same specimen. The IDO expression intensity was stronger in metastatic foci than in the primary tumor [15]. To date, however, the factors that play critical roles in sculpting the immunogenic phenotypes of PC cells remain elusive.

Studies have indicated that within the tumor microenvironment, Norepinephrine (NE) may be an important risk factor for metastasis in a variety of tumor types [16], [17]. First, by virtue of its presence in the circulation and its release by the sympathetic nervous system, NE is able to access organ systems throughout the body. Second, NE receptors are expressed in many organs, including the normal pancreas. In our previous study, we observed that various NE receptors were expressed on PC cells [18], [19]. Third, via its extracellular receptors and intracellular second messenger pathways, NE affects carcinogenesis. Studies have shown that sympathetic neurotransmitter signaling to the pancreas is responsible for biological and pathophysiological changes, mainly in the realms of endocrine and exocrine secretion, pancreatic blood and tumor progression [20]. Involvement of the NE system is consistent with an etiological role in cancer [17]. Catecholamines (CAs) play an additional role in the integration of signalling between the nervous system and the immune system. Recent evidence indicates that NE and epinephrine, through stimulation of theβ_2_-adrenoreceptor-cAMP-protein kinase A pathway, inhibit the production of type 1/proinflammatory cytokines, such as interleukin (IL-12), tumor necrosis factor-alpha, and interferon-gamma by antigen-presenting cells and T helper (Th) 1 cells, which causes a selective suppression of Th1 responses and cellular immunity [21], [22]. Either lowering the level of NE or blocking the NE receptors can reduce the probability of an individual developing cancer [23], [24]. Last but not least, some risk factors for PC progression can lead to aberrant increases in NE, such as cigarette smoking [25], mental stress [26], and diabetes mellitus [27].

The tumor immunosuppressive network is very complex [28], and this study intends to investigate the effects of Norepinephrine in immune evasion, including the direct effect and the synergistic effect with other critical factors. Gamma-interferon (IFN-γ) plays important roles in host immunoediting progression from immune surveillance to immune escape [29]. Furthermore, many studies indicated that IFN-γ could regulate MHC-I [30], [31], B7-1, IDO [32], [33] and B7-H1 [34], [35] expression in various cells.

Based on these findings, we hypothesize that NE could act to sculpt PC immune phenotypes by regulating MHC-I, B7-1, IDO and B7-H1 expression via the NE receptor pathway and allow PC cells to escape from the anti-tumor immune response.

## Methods

### Cell Lines and Culture Conditions

Two human PC cell lines, MIA PaCa-2 and BxPC-3, were purchased from American Type Culture Collection (Rockville, MD, USA). Cells were maintained in Dulbecco’s modified Eagle’s medium (DMEM) supplemented with 10% fetal bovine serum in 75 cm^2^ plastic flasks at 37°C in a 5% CO_2_/95% air atmosphere, and the medium was changed every 2–3 days. NE was purchased from Sigma-Aldrich (St. Louis, MO), as were the adrenergic receptor (AR) blockers, including prazosin (adrenergic alpha-1 antagonist), yohimbine (adrenergic alpha-2 antagonist), atenolol (adrenergic beta-1 antagonist) and butoxamine (adrenergic beta-2 antagonist). The day that the cells were seeded was counted as day 0. Cells (1×10^6^) were treated with norepinephrine (0 M, 10^−8^ M, 10^−7^ M, 10^−6^ M or 10^−5^ M), IFN-γ (0 ng/mL, 50 ng/mL, 100 ng/mL or 200 ng/mL) or adrenergic receptor blockers (10^−6^ M) in 5 mL of medium for 48 hours (h), 96 h, 1 week (w), 2 w, 3 w or 4 w. Cells were then used in assays measuring proliferation, invasion and mRNA expression as well as for immunocytochemistry and western blotting.

### Cell Proliferation Assay

The effect of NE on the growth of MIA PaCa-2 and BxPC-3 cells was determined using an MTT assay (OD 490 nm) as described previously [36]. All experiments had six replicates.

### The Membrane Invasion Culture System Assay

Transwell membrane invasion culture system chambers (8-µM pore size (Costar), previously described in [37]), were used to measure the invasiveness of the MIA PaCa-2 and BxPC-3 cells. After 24-hour incubation, the invading cells were stained with 0.1% crystal violet, counted by light microscopy and then diluted in 33% acetic acid. The number of invading cells was quantified using a FL 600 fluorescence plate reader (BioTek, Vermont, USA) at a wavelength of 570 nm. Experiments were performed in triplicate.

### Real-time Quantitative PCR Analysis

After total RNA was extracted and single-stranded cDNA was synthesized, real-time quantitative PCR analysis was conducted using the MJ Research DNA Engine OPTICON™ Continuous Fluorescence Detector system according to the manufacturer’s protocol. The sense primer 5′-TACGACGGCAAGGATTAC-3′ and the antisense primer 5′-TCCAGGTAGGCTCTCAAC-3′ were used for human MHC-I; the sense primer 5′-TCACTGGAG GGTCTTCTACG-3′ and the antisense primer 5′-GAGGTATGGACACTTGGATGG -3′ were used for human B7-1; the sense primer 5-TGACGCCTGTGTGAAAGC-3 and the antisense primer 5-AATCAGTGCCTCCAGTTCC-3 were used for human IDO; the sense primer 5′-GGTGGTGCCGACTACAAG-3′ and the antisense primer 5′-ATTGGTGGTGGTGGTCTTAC-3′ were used for human B7-H1. Quantitative normalization of cDNA in each sample was performed using the expression of human housekeeping gene glyceraldehyde-3-phosphate dehydrogenase (GAPDH; sense primer, 5′-TCATCCCTGCCTCTACTG-3′; antisense primer, 5′-TGCTTCACCACCTTCTTG-3′) as an internal control to determine the uniformity of the template RNA for all specimens. For each sample, MHC-I, B7-1, IDO and B7-H1 expression were derived from the ratio of their expression to GAPDH expression using the following formula: relative expression = 2^− (△Ct sample−△Ct calibrator)^.

### Western Blot Analysis

Primary antibodies for MHC-I (Santa Cruz Biotechnology, Inc.), B7-1 (Santa Cruz Biotechnology, Inc.), IDO (Chemicon International, Inc.) and B7-H1 (EBioscience, Inc.) were diluted 1∶400 in 5% nonfat dry milk powder with 0.2% PBS-Tween 20. Protein samples were solubilized and boiled in SDS sample buffer for 5 min and then separated using 12% SDS-PAGE. Next, the separated proteins were transferred to a polyvinylidene difluoride membrane. Following incubation in blocking buffer (5% nonfat dry milk powder with 0.2% PBS-Tween 20) for 1 h at room temperature and overnight incubation at 4°C with the primary antibodies, the membrane was washed and then probed with a horseradish peroxidase-linked secondary antibody (Dako Corp., Carpinteria, CA, USA; 1∶2,000) for 2 h at room temperature. After washing four times with PBS-Tween 20, the blot was developed with enhanced chemiluminescence substrate (Amersham Pharmacia Biotech, Piscataway, NJ, USA) and imaged using a Syngene Chemi-Genius imaging system (SynGene, UK).

### Immunocytochemistry (ICC)

MIA PaCa-2 and BxPC-3 cells from different experimental groups were cultured on 8 mm cover slips for 24 h. The cover slips were removed from the culture dishes for ICC staining [38]. Slide Scanner and Image Pro-Plus software were used to obtain the mean density of positive ICC staining (IOD/tumor tissue area), which represents the protein expression level.

### Statistical Analysis

Statistical analysis was conducted using SPSS software (Version 13.0, SPSS Inc., Chicago, IL, USA). Significant differences were determined by one-way analysis of variance. Values of *P*<0.05 were considered statistically significant.

## Results

### Dose-dependent Effects of NE/IFN-γ on Proliferation and Invasion

**Figure 1:**
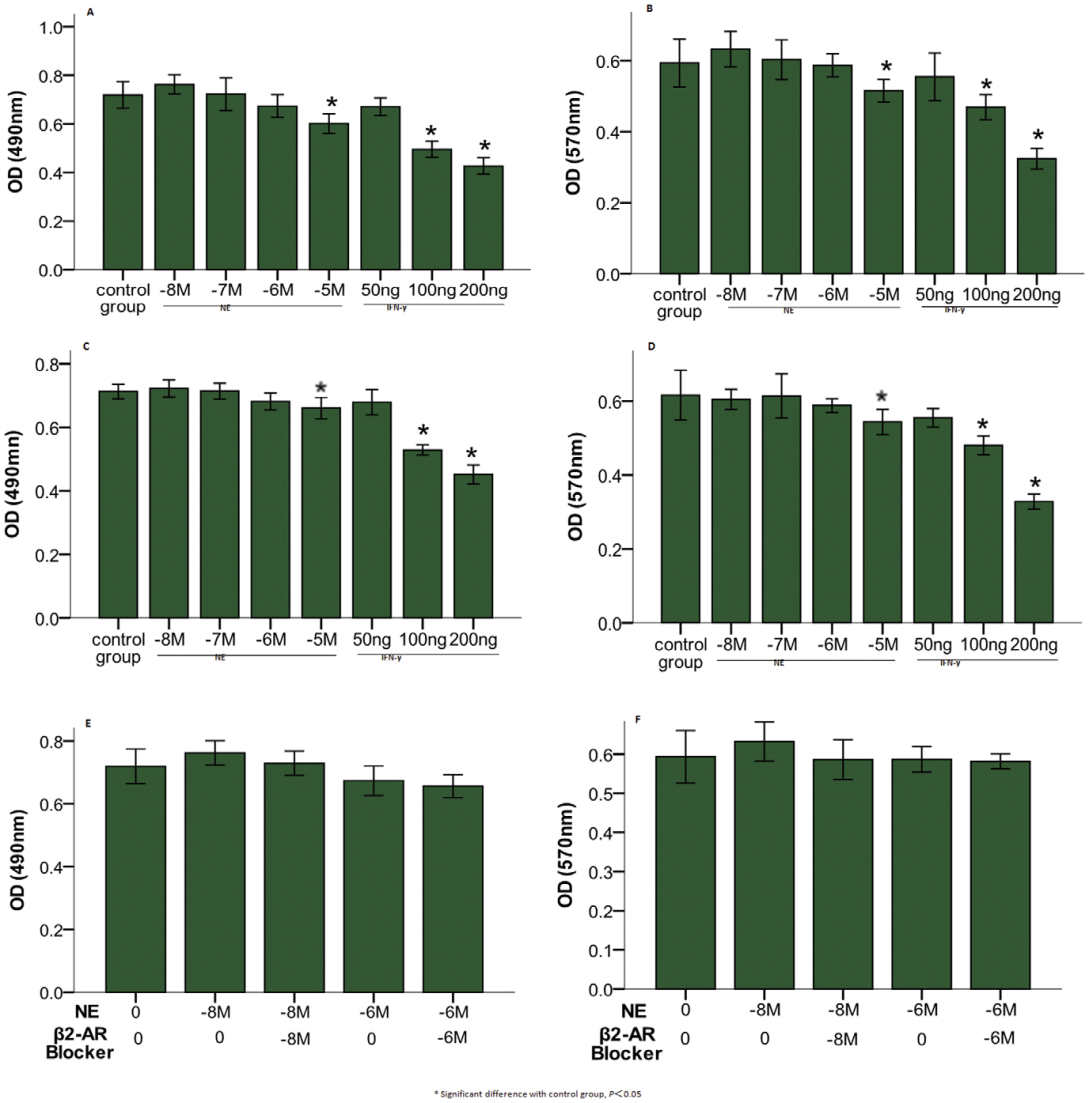
Dose-dependent effects of NE/IFN-γ on the proliferation and invasion of PC cells. NE and IFN-γ affect proliferation (OD 490 nm) and invasion (OD 570 nm) of MIA PaCa-2 (A and B) and BxPC-3 (C and D) cells in a dose-dependent manner. Low concentrations of NE (10^−8^ M) exhibited a trend toward stimulation, while NE (10^−6^ M) exhibited a trend toward suppression (*P*>0.05). High concentrations of NE (10^−5^ M) and IFN-γ (100 ng/mL, 200 ng/mL) had significant suppressive effects (*P*<0.05). A β_2_-AR antagonist decreased the stimulatory effects of NE (10^−8^ M) but did not alter the suppressive effects of NE (10^−6^ M) (E and F). (* Significant difference with control group, *P*<0.05).

Both NE and IFN-γ had dose-dependent effects on the proliferation and invasion of MIA PaCa-2 cells, and NE had bidirectional effects (Fig. 1A and 1B). NE (10^−8^ M) demonstrated a trend toward stimulation (*P*>0.05) and NE (10^−6^ M) toward suppression (*P*>0.05), whereas NE (10^−5^ M) had significant suppressive effects (*P*<0.05). IFN-γ (100 ng/mL and 200 ng/mL) exhibited significant suppressive effects (*P*<0.05). Similar effects of NE/IFN-γ were observed in BxPC-3 cells (Fig. 1C and 1D). When combined with the same concentration of the β_2_-AR antagonist butoxamine, the stimulatory effects of NE (10^−8^ M) decreased, and the suppressive effects of NE (10^−6^ M) did not change (Fig. 1E and 1F). NE and IFN-γ failed to have synergistic effects on proliferation and invasion in MIA PaCa-2 and BxPC-3 cell lines (*P*>0.05) (Fig. 1).

### Time-dependent Effects of NE/IFN-γ on Proliferation and Invasion

**Figure 2:**
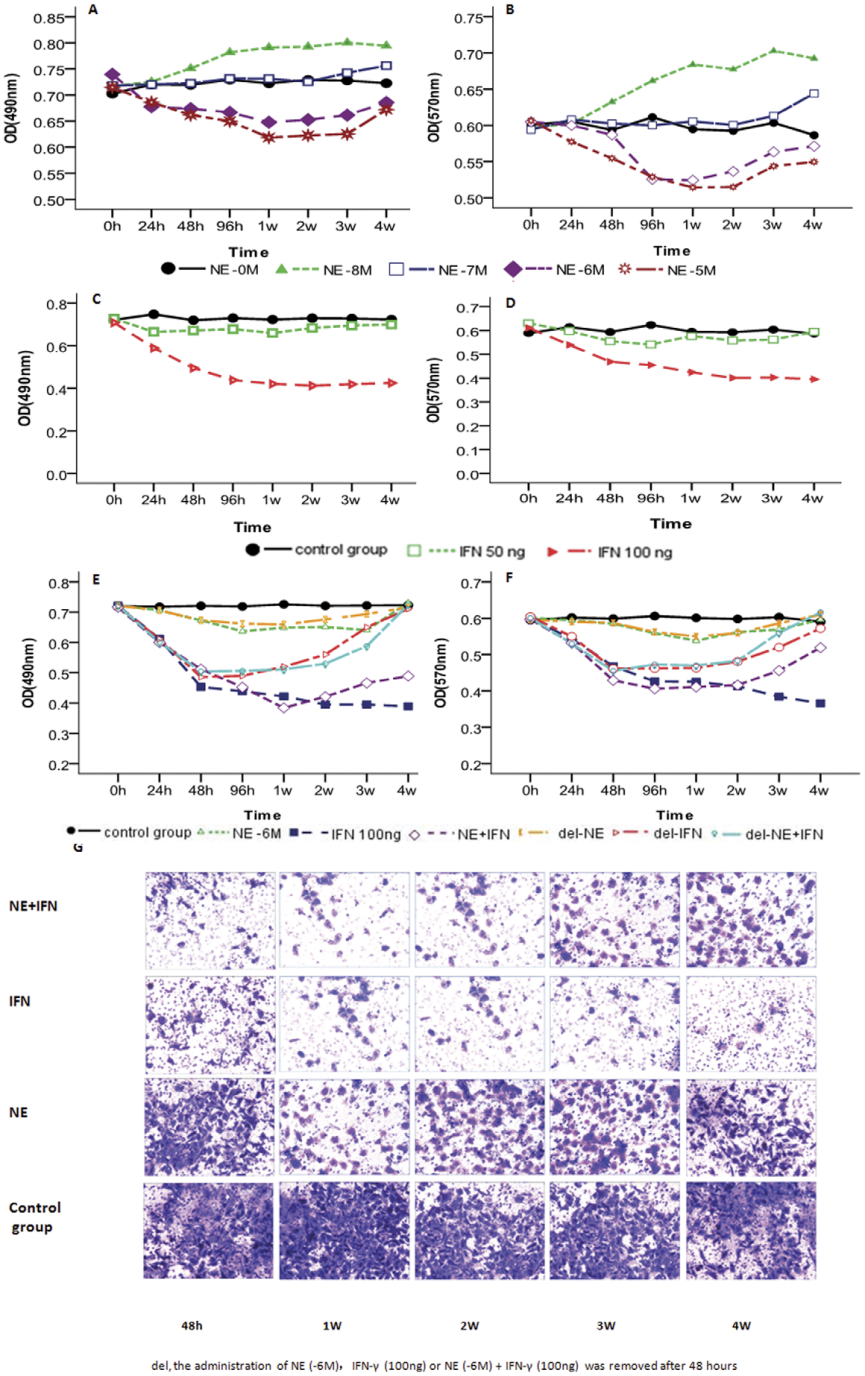
Time-dependent effects of NE/IFN-γ on cellular proliferation and invasion. The stimulatory effects of NE (10^−8^ M) continued to increase for 1 to 2 weeks, while the suppressive effects of NE (10^−6^ M, 10^−5^ M) were enhanced at 1 week and then were gradually reversed (A and B). The suppressive effects of IFN-γ (100 ng/mL) reached their peak at approximately 1 week and then stayed at the same level over the next 3 weeks (C and D). NE partially reversed the effects of IFN-γ (100 ng/mL) starting at 3 weeks. When NE (10^−6^ M), IFN-γ (100 ng/mL) or NE (10^−6^ M) plus IFN-γ (100 ng/mL) were removed, the suppressive effects began to decrease gradually starting at 2 weeks (E and F). The same phenomenon was also observed in the Transwell images (G), Magnification, 200×.

**Figure 3:**
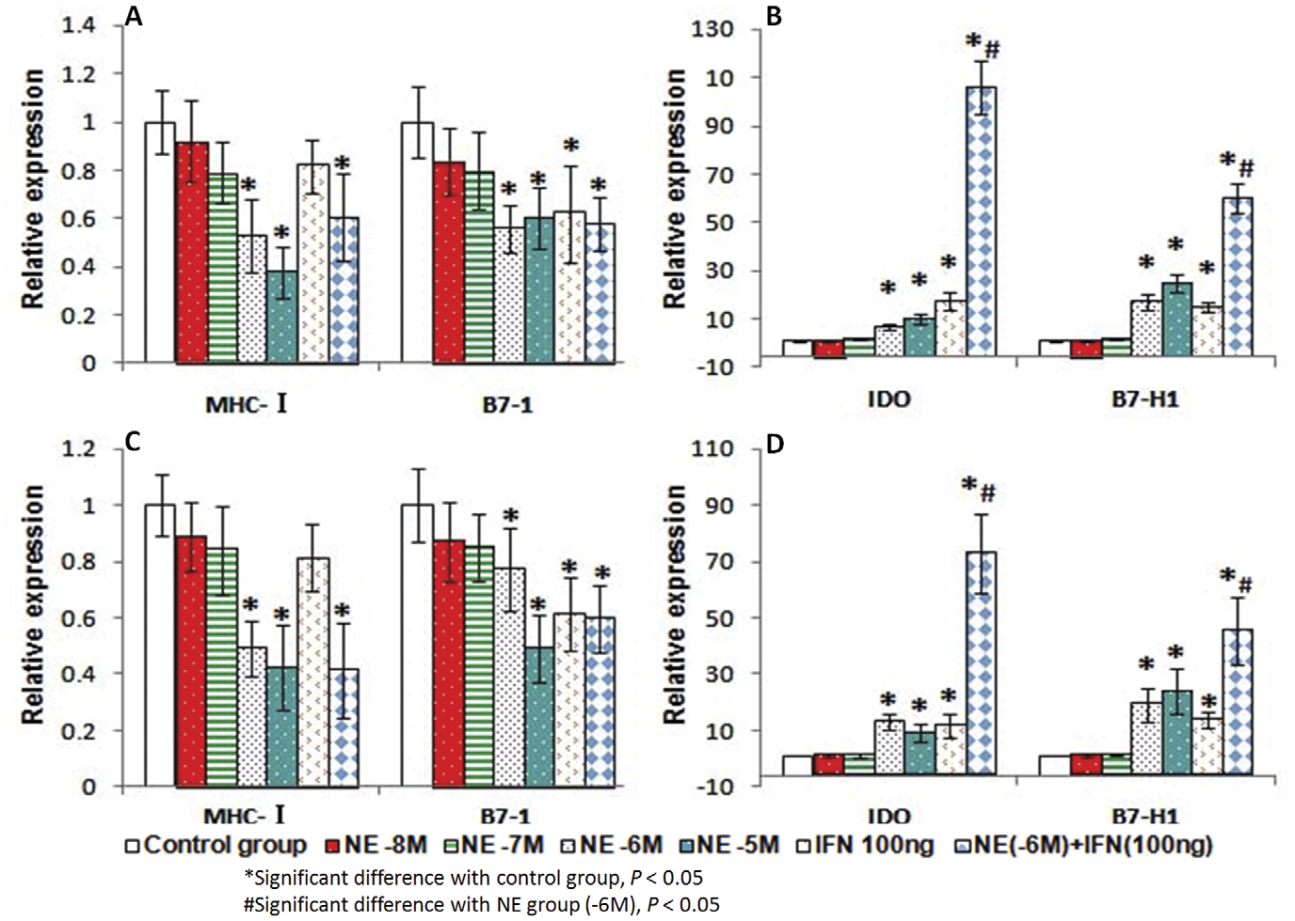
The effect of NE/IFN-γ on MHC-I, B7-1, IDO and B7-H1 expression in MIA PaCa-2 (A and B) and BxPC-3 cell lines (C and D). Compared with control group, NE suppressed MHC-I and B7-1 and up-regulated IDO and B7-H1 in a dose-dependent manner. IFN-γ suppressed B7-1, and up-regulated IDO and B7-H1. Compared with NE group, NE and IFN-γ had significant synergistic effects on IDO and B7-H1 (B and D).

NE had contradictory effects on PC cell proliferation and invasion depending on the concentration and duration of the treatment. The stimulatory effects of NE (10^−8^ M) continued to increase for 1 – 2 weeks, whereas the suppressive effects of NE (10^−6^ M and 10^−5^ M) increased over 1 week and then gradually reversed. NE (10^−7^ M) had no effects prior to week 4, at which point it began to exhibit stimulatory effects (Fig. 2A and B). Relative to the control and the 50 ng/mL IFN-γ treated groups, the 100 ng/mL IFN-γ treated group reached its peak at approximately 1 week (Fig. 2C and D) of treatment; this effect could be partially reversed by NE treatment through 3 weeks (Fig. 3E and F). When the NE (10^−6^ M), IFN-γ (100 ng/mL) or NE (10^−6^ M) plus IFN-γ (100 ng/mL) treatments were removed, the residual effects did not clear for 2 weeks, after which proliferation gradually increased (Fig. 2E and F). The changes in invasion were also shown in the Transwell images (Fig. 2G).

### Dose-dependent Effects of NE/IFN-γ on MHC-I, B7-1, IDO and B7-H1 Expression

NE had direct effects on MHC-I, B7-1, IDO and B7-H1 gene expression in the MIA PaCa-2 (Fig. 3A and B) and BxPC-3 cell lines (Fig. 3C and D). MHC-I, B7-1, IDO and B7-H1 expression were detectable in the control group, and NE treatment had significant, dose-dependent effects on expression. Increasing concentrations of NE suppressed MHC-I and B7-1 expression and significantly up-regulated IDO and B7-H1 expression (*P*<0.05). The combination of IFN-γ (100 ng/mL) and NE (10^−6^ M), had direct stimulatory and synergistic effects on IDO and B7-H1 expression (*P*<0.05) (Fig. 3B and D).

IFN-γ had no effects on modulating MHC-I expression either alone or in combination with NE (Fig. 3A and C). MHC-I expression in the 100 ng/mL IFN-γ treatment group was not down-regulated significantly compared to the control group (*P*>0.05). NE (10^−6^ M) plus IFN-γ (100 ng/mL) did not significantly further decrease MHC-I expression relative to treatment with 10^−6^ M NE (*P*>0.05).

### The Target Receptors for NE/IFN-γ-mediated Effects on MHC-I, B7-1, IDO and B7-H1 Expression

**Figure 4:**
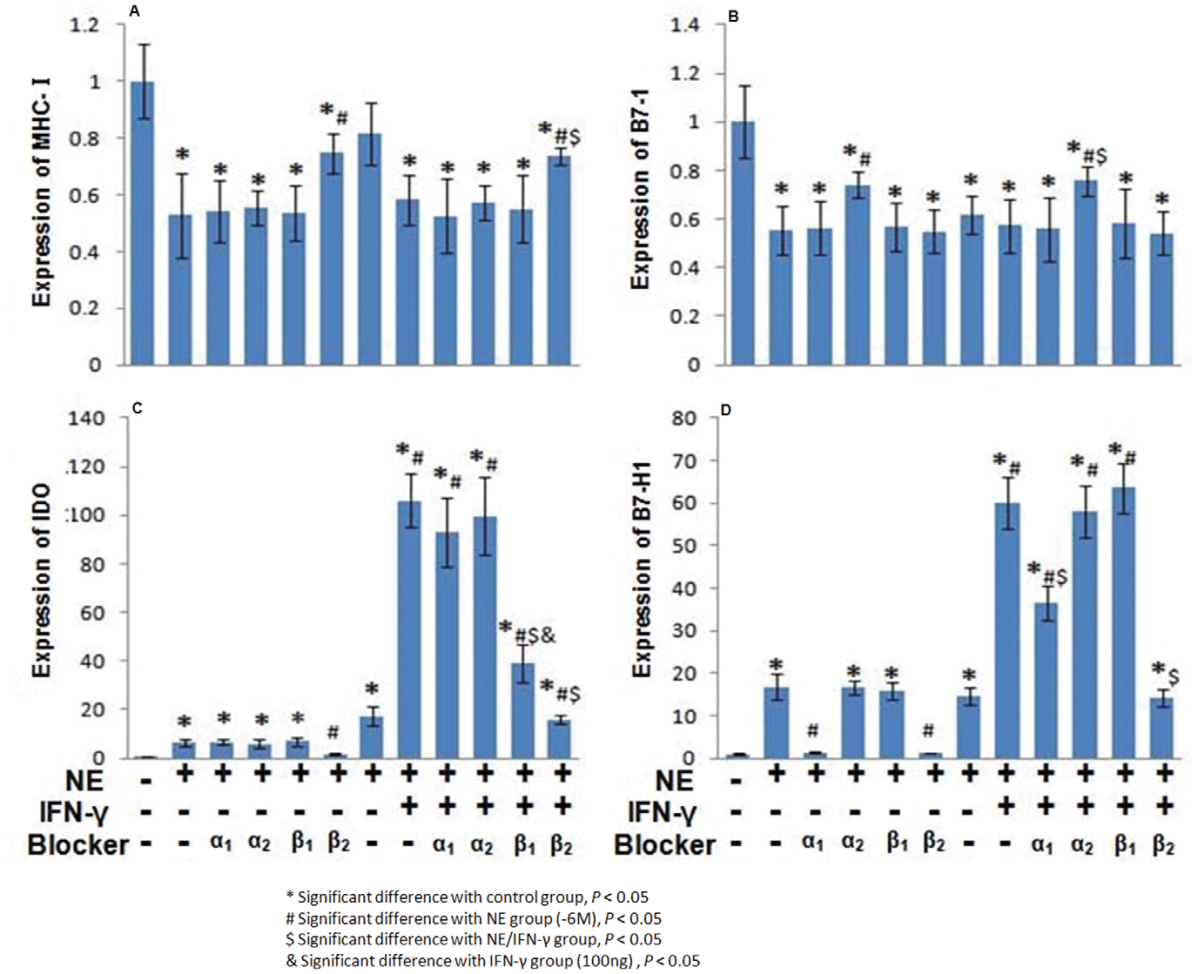
The receptors by which NE and IFN-γ affect MHC-I, B7-1, IDO and B7-H1 expression. NE exerted its effects on MHC-I (A), IDO (C) and B7-H1 (D) expression mainly through the β_2_ adrenergic receptor and on B7-1 (B) through the α_2_ adrenergic receptor. (* Significant difference from control group, P<0.05; # Significant difference from NE group (10^−6^ M), P<0.05; $ Significant difference from NE/IFN-γ group, P<0.05; & Significant difference from IFN-γ group (100 ng/mL), P<0.05).

**Figure 5:**
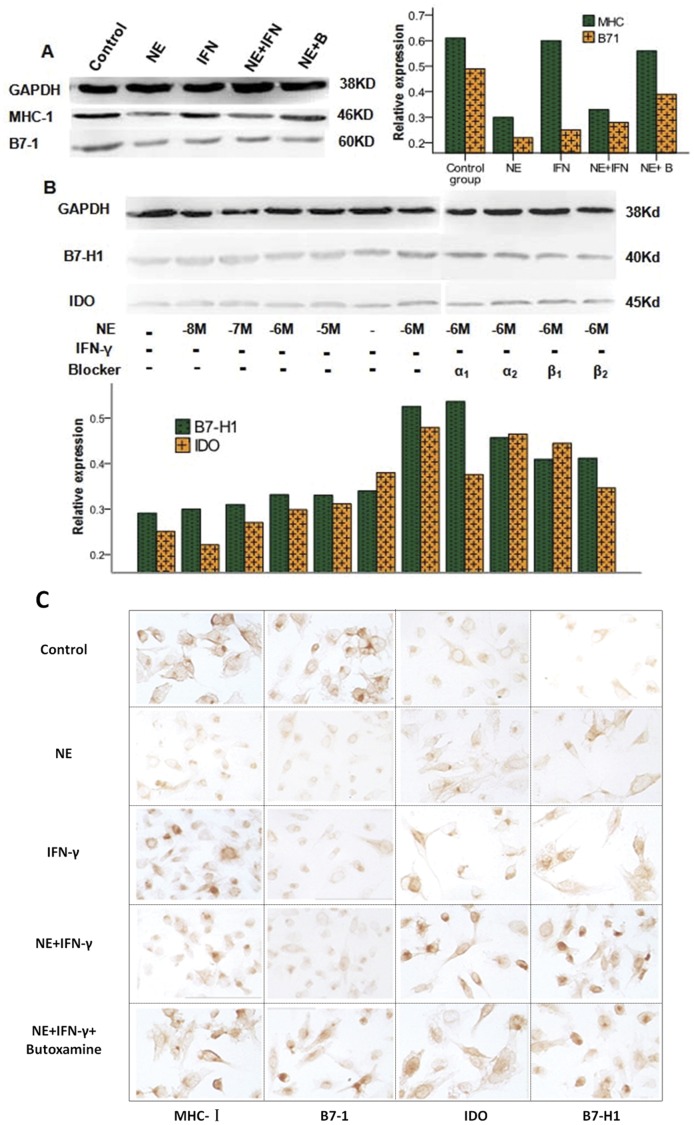
The effects of NE/IFN-γ on MHC-I, B7-1, IDO and B7-H1 protein expression. Western blot results are shown on the left and at the top; histograms depicting relative intensities are shown on the right and at the bottom (A and B). Immunohistochemical staining of MIA PaCa-2 cells is shown at 400×magnification (C).

NE exerted its direct and synergistic effects on MHC-I, B7-1, IDO and B7-H1 expression mainly through β_2_-AR. The direct effects and synergistic effects of NE on MHC-I expression could be partially blocked by butoxamine (a β_2_-AR blocker) (Fig. 4A), and the effects of NE on B7-1 expression were partially blocked by yohimbine (an α_2_-AR blocker) (Fig. 4B). The direct and synergistic effects of NE on IDO expression could be completely reversed by butoxamine, and the synergistic effects of NE were partially reduced by atenolol (a β_1_-AR blocker) (Fig. 4C). Both butoxamine and prazosin (an α_1_-AR blocker) could completely block the direct effects of NE on B7-H1 expression, and its synergistic effects could be completely blocked by butoxamine and partially by prazosin (Fig. 4D). NE had similar direct and synergistic effects on MHC-I, B7-1, IDO and B7-H1 protein expression and mRNA expression (Fig. 5).

### Time-dependent Effects of NE/IFN-γ on MHC-I, B7-1, IDO and B7-H1 Expression

**Figure 6:**
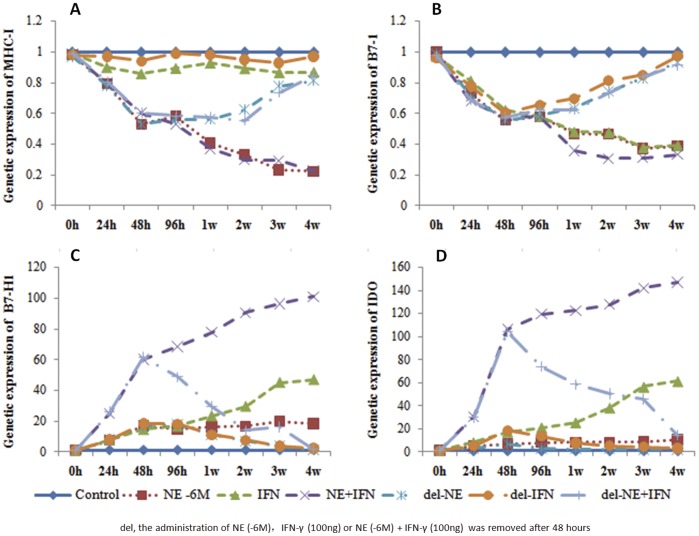
Time-dependent effects of NE/IFN-γ on MHC-I (A), B7-1 (B), IDO (C) and B7-H1 (D) mRNA expression. NE/IFN-γ had time-dependent suppressive effects on MHCI and B7-1 and stimulatory effects on IDO and B7-H1 expression. When NE (10^−6^ M), IFN-γ (100 ng/mL) or NE (10^−6^ M) plus IFN-γ (100 ng/mL) was removed after 48 hours, the residual effects decreased dramatically within 1 week.

NE/IFN-γ exhibited time-dependent suppressive effects on MHC-1 and B7-1 expression and stimulatory effects on IDO and B7-H1 expression (Fig. 6). With increased treatment duration, the expression levels of MHC-I, B7-1, IDO and B7-H1 were unchanged in the control group. NE (10^−6^ M) did not exhibit time-dependent effects on either IDO or B7-H1 expression (*P*>0.05) but was able to suppress MHC-1 and B7-1 expression (*P*<0.05). IFN-γ (100 ng/mL) demonstrated significant inhibitory effects on B7-1 expression in a time-dependent manner and exhibited stimulatory effects on IDO and B7-H1 expression starting at 1 and 2 weeks (*P*<0.05). NE (10^−6^ M) plus IFN-γ (100 ng/mL) treatment had significant time-dependent inhibitory effects on MHC-I and B7-1 expression and stimulatory effects on IDO and B7-H1 expression (*P*<0.05). When the cells were treated with NE (10^−6^ M), IFN-γ (100 ng/mL) or NE (10^−6^ M) plus IFN-γ (100 ng/mL) for 48 hours and then left untreated for 1 week, these effects all decreased dramatically.

## Discussion

Previous studies have indicated that tumor prognosis is closely associated with immune escape by tumor cells [39]. MHC-1 and B7-1 play important roles in tumor progression and affect the outcome of immunotherapy [40], [41]. Additionally, IDO and B7-H1 play key roles in immune tolerance and tumor immunoediting [12], [42]. NE, either on its own or in combination with IFN-γ, may sculpt the PC cell immune phenotype by modulating MHC-1, B7-1, IDO and B7-H1 expression via adrenergic receptors (ARs).

NE exhibits inconsistent effects on the biological properties of tumor cells. Borresen [43] suggested that the depletion of NE in normal tissues that neighbor tumors may be important for invasion and accelerated malignant growth. Similarly, Bastian [44] observed a reduced migratory activity after NE administration. However, more recent studies support the idea that NE is an etiological factor in some types of cancer [17], and increased NE can stimulate the proliferation or invasion of tumor cells via β-ARs. The proliferation and migration of the human PC cell lines Panc-1, BxPC-3 and HPDE6-c7 are also regulated by β-ARs via cAMP-dependent signaling [45]. In this study, we observed that NE had significant dose-dependent bidirectional effects on the proliferation and invasion of both MIA PaCa-2 and BxPC-3 cells at 48 h in vitro. Low concentrations of NE (10^−8^ M) demonstrated a trend toward stimulation, NE (10^−6^ M) exhibited a suppressive trend, and a high concentration of NE (10^−5^ M) suppressed proliferation and invasion significantly. Further, the stimulatory effects of NE (10^−8^ M) could be reversed with β_2_-AR antagonists, while the suppressive effects of NE (10^−6^ M) could not. Therefore, we suggest that the suppressive effect of high concentration NE treatment on PC cells was only a consequence of the inability of these cells to adapt to altered culture conditions. The stimulatory effects of low concentrations of NE may mimic its biological functions.

Time-dependent effects of treatments with various concentrations of NE were further investigated, for lengths of up to 4 weeks. The results showed that the stimulatory effects of NE (10^−8^ M) increased gradually during the first 1 to 2 weeks and then plateaued. The suppressive effects of 10^−6^ M and 10^−7^ M NE dosages were also enhanced at 1 week but declined at 2 weeks and nearly disappeared at 4 weeks. These results support our hypothesis that each of the different concentrations of NE used would all have stimulatory effects on PC cell proliferation and invasion over the long term.

We also observed that IFN-γ had suppressive effects on PC cell proliferation and invasion. Furthermore, we noticed that NE could prevent the suppression of growth and invasion induced by IFN-γ when co-administered at 10^−6^ M for 2 weeks, perhaps because the NE effects were dominant over long time periods. However, this effect is not sufficiently pronounced to allow PC cell progression in an immunocompetent host. NE (by itself or in combination with IFN-γ) may play a vital role in sculpting the immunogenic phenotype of PC, starting a “cancer immunoediting” process.

Aberrant tumor-associated expression patterns of MHC-1, B7-1, IDO and B7-H1 play a crucial role in immune evasion. Previous studies indicate that IFN-γ markedly up-regulates transcription of genes involved in MHC-1 assembly [46] via a cis-acting regulatory element site of the MHC class I promoter. Moreover, IFN-γ modulates B7-1 expression in various cells via the MAPK pathway, in which a distinct B7-1-responsive element corresponding to the IFN regulatory factor-7 (IRF-7) binding site identified in the B7-1 promoter plays a vital role [47]. In our study, low expression of MHC-1 and B7-1 were observed in PC cells. However, IFN-γ did not promote, but suppressed B7-1 expression and had no effect on MHC-1 expression. Current studies indicate that IFN-γ can upregulate MHC-1 expression of many kinds of tumor cells [48], [49]. But we did not find relative studies in pancreatic carcinoma. Therefore, we repeated this experiment with positive control of human leukemia cell line U937 and human hepatoma cell HepG2 and same results were observed. In our study, we also observed that IFN-γ could up-regulate IDO and B7-H1 expression. Studies have indicated that IFN-γ modulates B7-H1 expression via the MEK/ERK pathway and that inhibition of STAT1 reduces B7-H1 expression [50]. IRF-1 is primarily responsible for constitutive B7-H1 expression and for the IFN-γ-mediated B7-H1 up-regulation [51]. The up-regulation of IDO induced by IFN-γ involves two IFN-stimulated response elements and six regulatory sequences in the IDO promoter that are similar to the γ-IRF binding sequence [52].

Previous studies indicated that stress and depression, which were related to the level of NE [26], could impair the anti-tumor immune response and contribute to development and progression of some types of cancer [20], [53]. We observed dose-dependent and time-dependent effects of NE on MHC-1, B7-1, IDO and B7-H1 expression. High concentrations of NE significantly changed the expression of these factors, whereas low concentrations did not, at least in the short term. High concentrations of NE suppressed MHC-1 and B7-1 expression and up-regulated IDO and B7-H1 expression but suppressed proliferation and invasion at short time points; however, low concentrations of NE acted to modulate expression and biological readouts simultaneously, also at long time points. Therefore, long-term NE intervention might provide more opportunities for immune escape and PC progression.

Importantly, NE had significant synergistic effects with IFN-γ on both IDO and B7-H1 expression. These effects may be due to the activation of the cAMP/PKA and MAPK/ERK1/2 signaling pathway by both β_2_-AR [54] and IFN-γ [55]. Moreover, NE could up-regulate IFN-γ receptors expression resulting in IDO activation, as some pro-inflammatory cytokines do [56].

We observed in this study that NE impacted PC immune phenotypes in vitro mainly via β_2_-AR. This finding suggests that interventions targeting components of the activated sympathetic-adrenal medullary axis or the utilization of β-AR blocking agents may represent new strategies for slowing down the progression of this malignant disease and improving patients’ quality of life [57].

Interestingly, we observed that when 10^−6^ M NE or the combination of NE and IFN-γ was administered for 48 hours and then removed, the residual effects on PC cell proliferation and invasion lasted for approximately 1 to 2 weeks, while the effects on gene expression decreased rapidly. Both NE [58] and IFN-γ [59] can induce tumor cell apoptosis and suppress proliferation at short time points, which may explain why the up-regulation of IDO and B7-H1 decreased rapidly. Short-term NE hampered rather than accelerated proliferation.

### Conclusions

NE plays an important role in modulating PC cell proliferation and invasion as well as in sculpting the immune phenotype by regulating MHC-1, B7-1, IDO and B7-H1 expression in vitro. These effects are dependent on the NE concentration, the presence of other molecules and time, among other variables. At short time points, NE is not able to impact PC cell differentiation, but it can increase the expression levels of IDO and B7-H1, allowing PC cells to defend against host immune attacks, and over time, to evade the immune response. Time is clearly an important factor in the relationship between conditions that result in the chronic up-regulation of NE and PC progression.
